# Bromethalin Exposure in Dogs and Cats: A 14‐Year Retrospective Study (2010–2023) From the California Animal Health and Food Safety Laboratory System

**DOI:** 10.1111/jvim.70057

**Published:** 2025-03-26

**Authors:** Sigal Klainbart, Marcos Pérez‐López, Michael S. Filigenzi, Robert H. Poppenga

**Affiliations:** ^1^ California Animal Health and Food Safety Laboratory System University of California, Davis California USA; ^2^ Department of Small Animal Emergency and Critical Care The Veterinary Teaching Hospital, Koret School of Veterinary Medicine, The Hebrew University of Jerusalem Jerusalem Israel; ^3^ Toxicology Unit Faculty of Veterinary Medicine Universidad de Extremadura Cáceres Spain

**Keywords:** convulsant syndrome, desmethylbromethalin, liquid chromatography‐mass spectrometry, paralytic syndrome, rodenticide

## Abstract

**Background:**

Bromethalin, a rodenticide, is increasingly used due to restrictions on other rodenticides.

**Objectives:**

The study aimed to analyze the frequency, demographics, clinical signs, and diagnostics of suspected bromethalin intoxication in dogs and cats.

**Animals:**

Two hundred twenty‐three cases (249 samples) involving 123 dogs and 100 cats suspected or confirmed to have bromethalin intoxication were submitted to the toxicology laboratory at the California Animal Health and Food Safety Laboratory.

**Methods:**

This was a retrospective cohort study. Between 2010 and 2023, Liquid Chromatography–Mass Spectrometry was used to detect desmethylbromethalin (DMB), bromethalin's metabolite, in various tissues and serum.

**Results:**

Cases increased 2.8‐fold from 59 (2010–2016) to 164 (2017–2023). Cats were significantly younger (median 24 months, IQR: 41.5) than dogs (36 months, IQR: 60.0; *p* = 0.016) and were more likely to have confirmed DMB exposure (60% vs. 25%, *p* < 0.0001). Submitted samples for analysis were adipose tissue (37%), liver (20%), and brain (19%). Clinical signs included seizures, tremors, weakness, and paralysis. Magnetic resonance imaging (MRI) findings in 17 dogs and cats were consistent with bromethalin intoxication in 77% of cases. Autopsies (33 cases) revealed CNS lesions compatible with bromethalin toxicosis in 2/8 dogs and in 24/25 cats.

**Conclusions and Clinical Importance:**

Bromethalin exposure is increasingly prevalent in pets. Adipose tissue remains the most reliable sample for diagnosis; cats are more likely to test positive for DMB and exhibit compatible autopsy results. MRI findings can also support the diagnosis. These insights could assist in diagnosing and managing bromethalin intoxication in pets.

**Abbreviations**
ADC: apparent diffusion coefficient
AR: anticoagulant rodenticide
ATP: adenosine 5′‐triphosphate
CAHFS: California Animal Health and Food Safety Laboratory System
CNS: central nervous system
CSF: cerebrospinal fluid
DMB: desmethylbromethalin
DWI: diffusion‐weighted imaging
EEG: electroencephalogram
EPA: Environmental Protection Agency
EU: European Union
LC–MS/MS: liquid Chromatography‐Mass Spectrometry
LD_50_
: lethal dose 50%
MRI: magnetic resonance imaging
US: United States

## 1 Introduction

**FIGURE 1:**
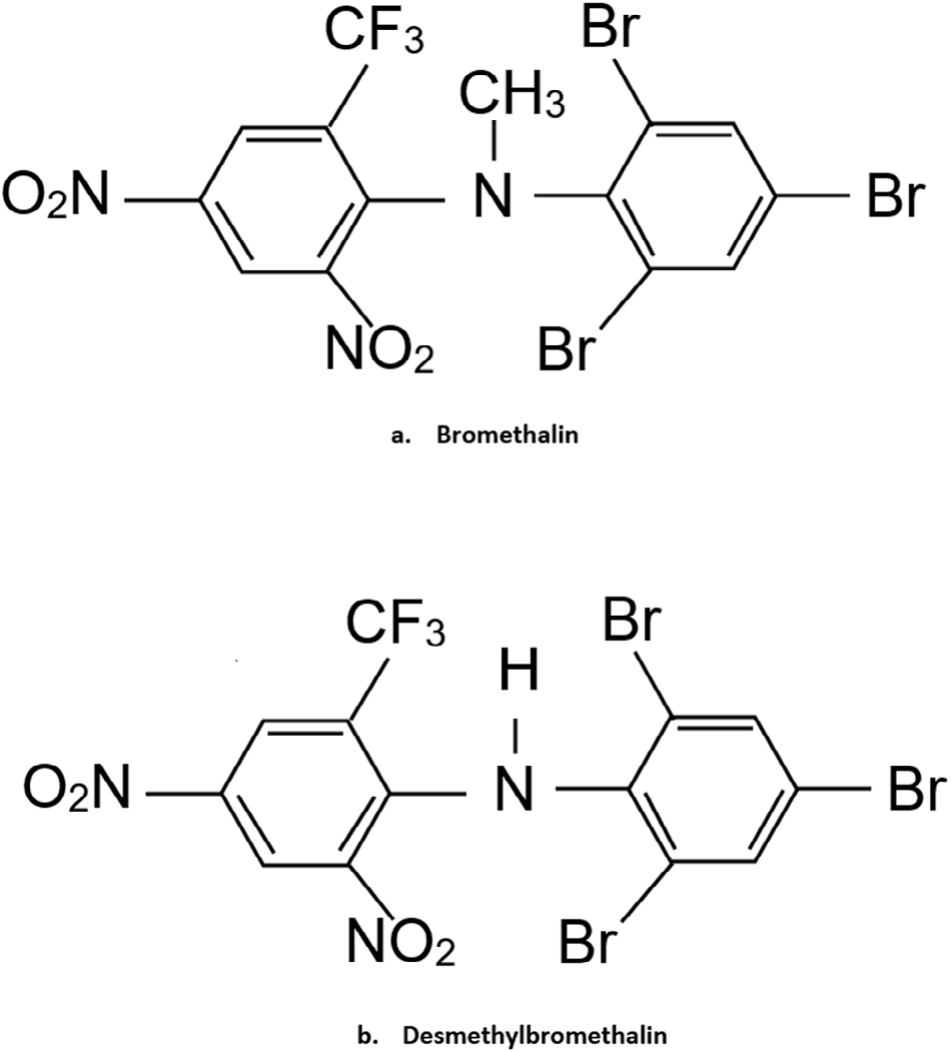
Chemical structure of bromethalin (a) and its metabolite desmethylbromethalin (DMB; b).

Rodenticides are commonly used to control pest populations, with bromethalin being a widely utilized acute, single‐feeding rodenticide. Discovered in the 1970s and introduced in the U.S. in 1985 [1], bromethalin was developed to combat warfarin‐resistant rodents. In 2011, the Environmental Protection Agency (EPA) restricted the residential use of second‐generation anticoagulant rodenticides (ARs) due to poisoning risks in children, pets, and wildlife, leading to increased sales of bromethalin products [2, 3]. In contrast, bromethalin is banned in the European Union [4]. It is marketed in formulations containing 0.01%–0.025% (0.1–0.25 mg/g) and must be used in bait boxes or underground [5]. Chemically, it is a diphenylamine compound [*N*‐methyl‐2,4‐dinitro‐*N*‐(2,4,6‐tribromophenyl)‐6‐(trifluoromethyl) benzylamine, C12H17Br3F3N3O4], (Figure 1a) [5]. After ingestion, bromethalin is rapidly absorbed, reaching peak plasma concentrations within 4–6 h. It is metabolized in the liver by cytochrome P450 to desmethylbromethalin (DMB), a metabolite as or more toxic than the parent compound (Figure 1b) [6]. The lethal dose 50% (LD50) ranges from 2.4 to 5.6 mg/kg in dogs and 0.4 to 0.7 mg/kg in cats, with fatalities reported at lower doses [7, 8]. Species with limited *N*‐demethylase activity, such as guinea pigs, exhibit resistance [3]. Bromethalin has a plasma half‐life exceeding 3–6 days and distributes extensively in body tissues, particularly fat [9]. Excretion occurs primarily via bile, with enterohepatic circulation and minimal renal elimination [9]. Bromethalin and DMB act as potent oxidative phosphorylation uncouplers in the central nervous system (CNS), leading to ATP depletion, ion pump dysfunction, and lipid peroxidation.6 This results in myelin sheath edema, axonal damage, and increased intracranial pressure [6, 7, 10, 11]. Clinical signs appear within 10 h to several days and persist for up to 12 days [5]. Two distinct syndromes are observed: the “paralytic syndrome” in sublethal exposures, characterized by ataxia, tremors, and hind limb paresis, and the “convulsant syndrome” in higher exposures, leading to seizures and rapid deterioration [7, 8, 11, 12, 13, 14]. Cats are more susceptible and predominantly develop the paralytic syndrome [8]. Juveniles exhibit increased sensitivity [11]. The risk of secondary toxicosis in pets from consuming bromethalin‐affected prey is low but theoretically possible in cats [11]. Diagnosis is based on exposure history, clinical presentation, and imaging findings, including MRI‐detected diffuse leukoencephalopathy [15, 16]. Histopathological findings include cerebral edema and spongy degeneration of CNS white matter [7, 8, 9, 13]. Liquid chromatography–mass spectrometry (LC–MS/MS) can detect DMB in serum, plasma, and tissues [9, 13, 17]. While bromethalin toxicosis is well documented in laboratory settings, its effects in a large clinical population of pets remain understudied. This retrospective study aims to describe suspected and confirmed cases of bromethalin exposure in dogs and cats from the Toxicology Section at the California Animal Health and Food Safety Laboratory.

## 2 Materials and Methods

### 2.1 Analytical Method for Detection of DMB

The analysis to detect bromethalin exposure utilized at the toxicology laboratory of the California Animal Health and Food Safety Laboratory is based on a published and validated method and relies on the detection of the metabolite, DMB, in tissues or serum by LC–MS–MS [17]. (The method description can be found in a supplemental file).

The selection of DMB, rather than bromethalin, as the analyte for detection is based on several key factors: bromethalin is rapidly absorbed after ingestion and metabolized in the liver via *N*‐demethylation into DMB in most species, making it a reliable indicator of bromethalin exposure. Additionally, testing for DMB confirms both the ingestion and metabolism of the parent compound, with analyzes primarily providing qualitative results (positive or negative). Furthermore, bromethalin exhibits poor responsiveness to electrospray LC–MS, rendering it unsuitable for low‐level detection in tissue samples, while DMB remains consistently detectable and more reliable for diagnostic purposes [17].

### 2.2 Data Collection

For each received sample, data was collected, to the best extent possible, from the submission form or through a phone call. Available data typically includes information about the exposed species, gender, age, specimen collection location, a brief medical history, suspected or observed exposure, observed clinical effects, treatments administered, MRI results, outcome, and post‐mortem examination results.

### 2.3 Statistical Analysis

The distribution pattern of quantitative variables was assessed using the Shapiro–Wilk test. Quantitative variables were compared between groups using the Student's *t*‐test or Mann–Whitney *U*‐test, for normally and non‐normally distributed variables, respectively. Categorical variables were compared between groups using the chi‐squared or Fisher's exact tests, as appropriate. *p* < 0.05 was considered significant. Analyzes were performed using a statistical software package (SPSS 22.0 for Windows, IBM Corp., Armonk, N.Y., USA).

## 3 Results

**FIGURE 2:**
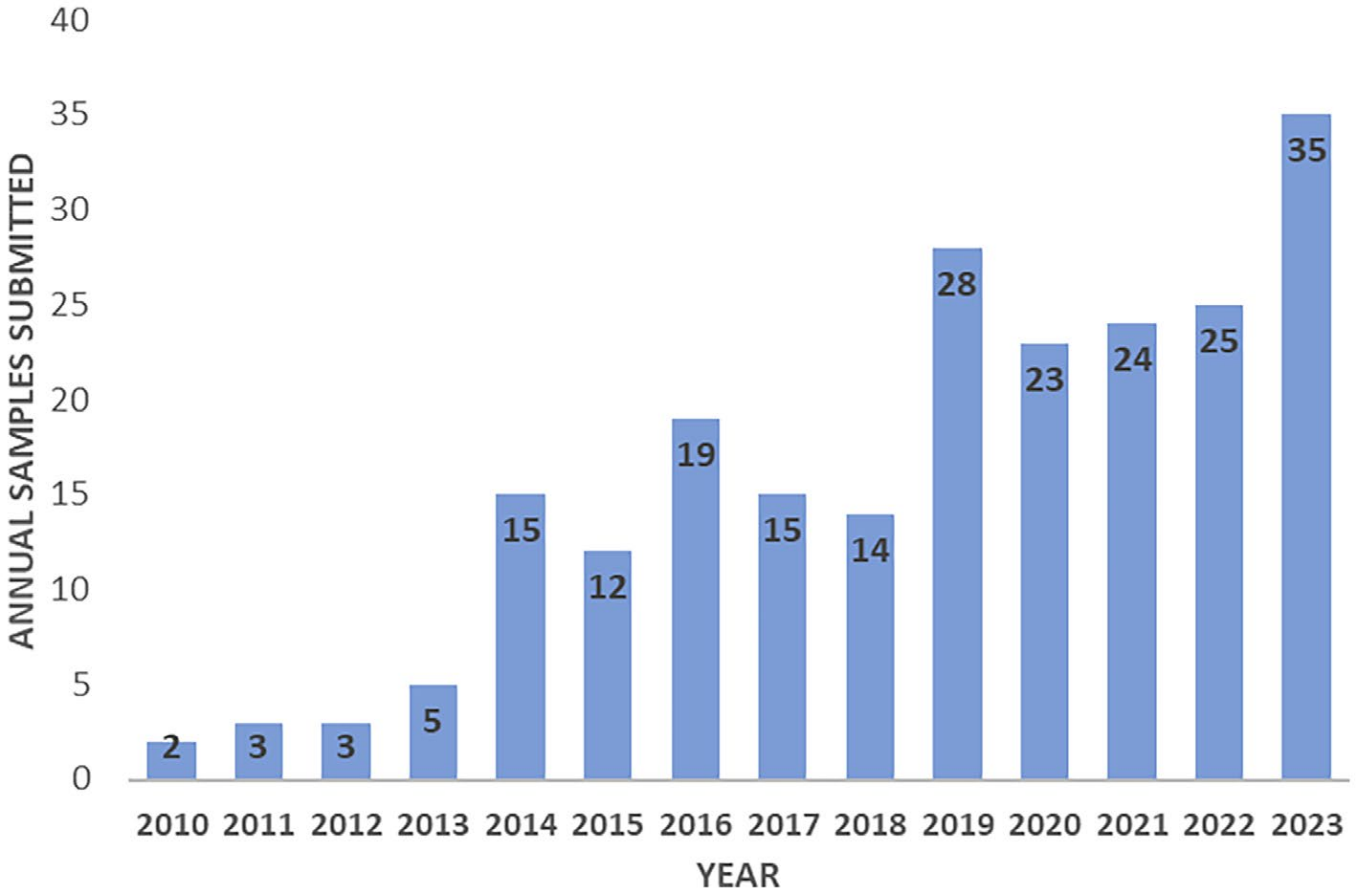
A total of 223 canine and feline cases were submitted to the toxicology laboratory at the California Animal Health and Food Safety Laboratory System for bromethalin analysis between 2010 and 2023, divided by years. The number of submitted cases increased 2.8‐fold, from 59 in 2010–2016 to 164 cases in 2017–2023.

**FIGURE 3:**
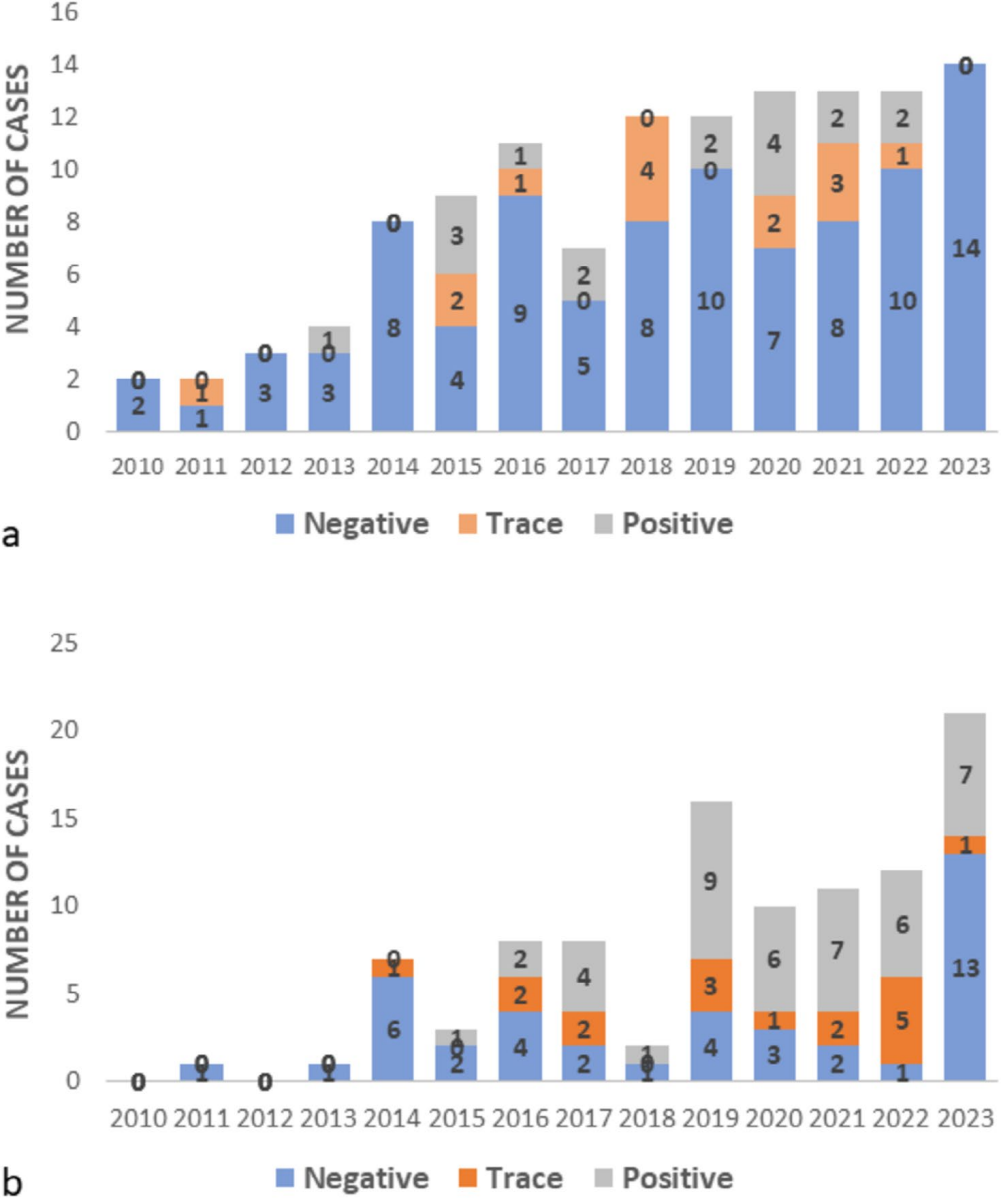
Bromethalin analysis results, divided by years (2017–2023), in dogs (a) and cats (b).

**FIGURE 4:**
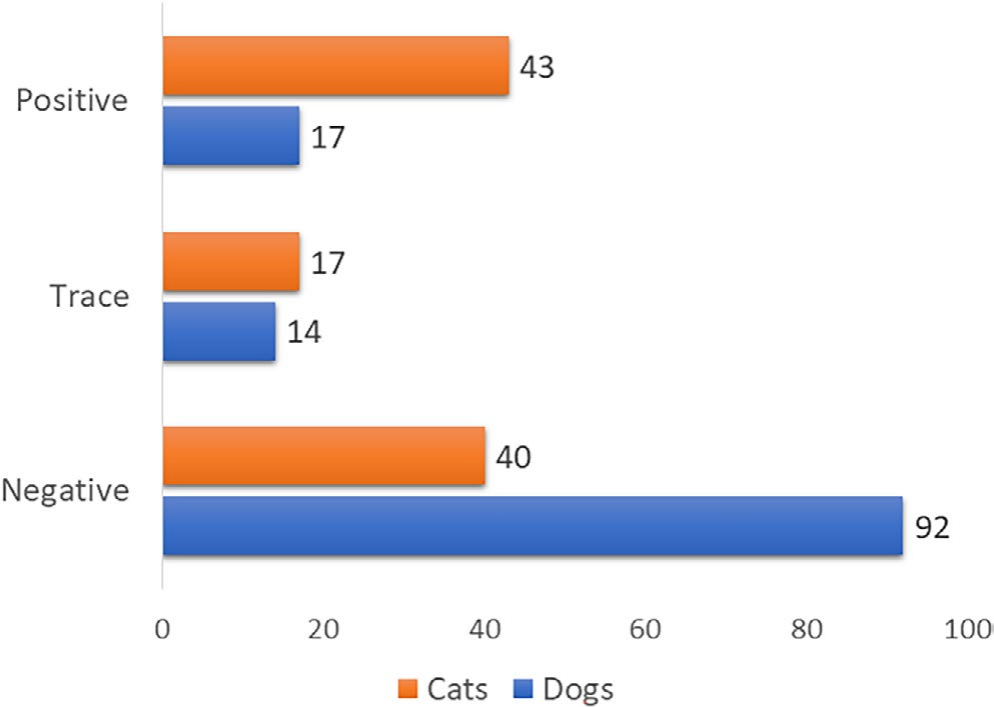
Overall positive, trace, and negative bromethalin analysis results, divided by species.

Between January 1st, 2010, and December 31st, 2023, 123 canine and 100 feline cases, including 249 samples (some cases included more than one specimen), were submitted to the toxicology laboratory at the California Animal Health and Food Safety Laboratory for bromethalin analysis from 35 states (Figure 2). The number of submitted cases increased 2.8‐fold, from 59 in 2010–2016 to 164 cases in 2017–2023. Of the 99 dogs for which information was available, 53 were female (20 spayed) and 46 were male (20 neutered); the median age of 88 dogs with available information was 36 months (range: 1.75–180 months, interquartile range [IQR]: 60.0 months). Of the 79 cats for which information was available, 41 were female (12 spayed) and 38 were male (11 neutered); the median age of 73 cats with available information was 24 months (range: 0.75–168 months, IQR: 41.5 months). Cats were significantly younger than dogs (*p* = 0.016). Overall, 132/223 (59%) cases were negative, 31 (14%) had a trace amount of DMB, and 60 (27%) were positive (at least in one sample). When divided by species, 92/123 (75%) dogs were negative, 14 (11%) had a trace amount of DMB, and 17 (14%) were positive. In contrast, 40/100 (40%) cats were negative, 17 (17%) had a trace amount of DMB, and 43 (43%) were positive. Cats were significantly more likely to have evidence (trace + positive) of DMB in their sample than dogs (*p* < 0.0001; Figures 3 and 4).

**FIGURE 5:**
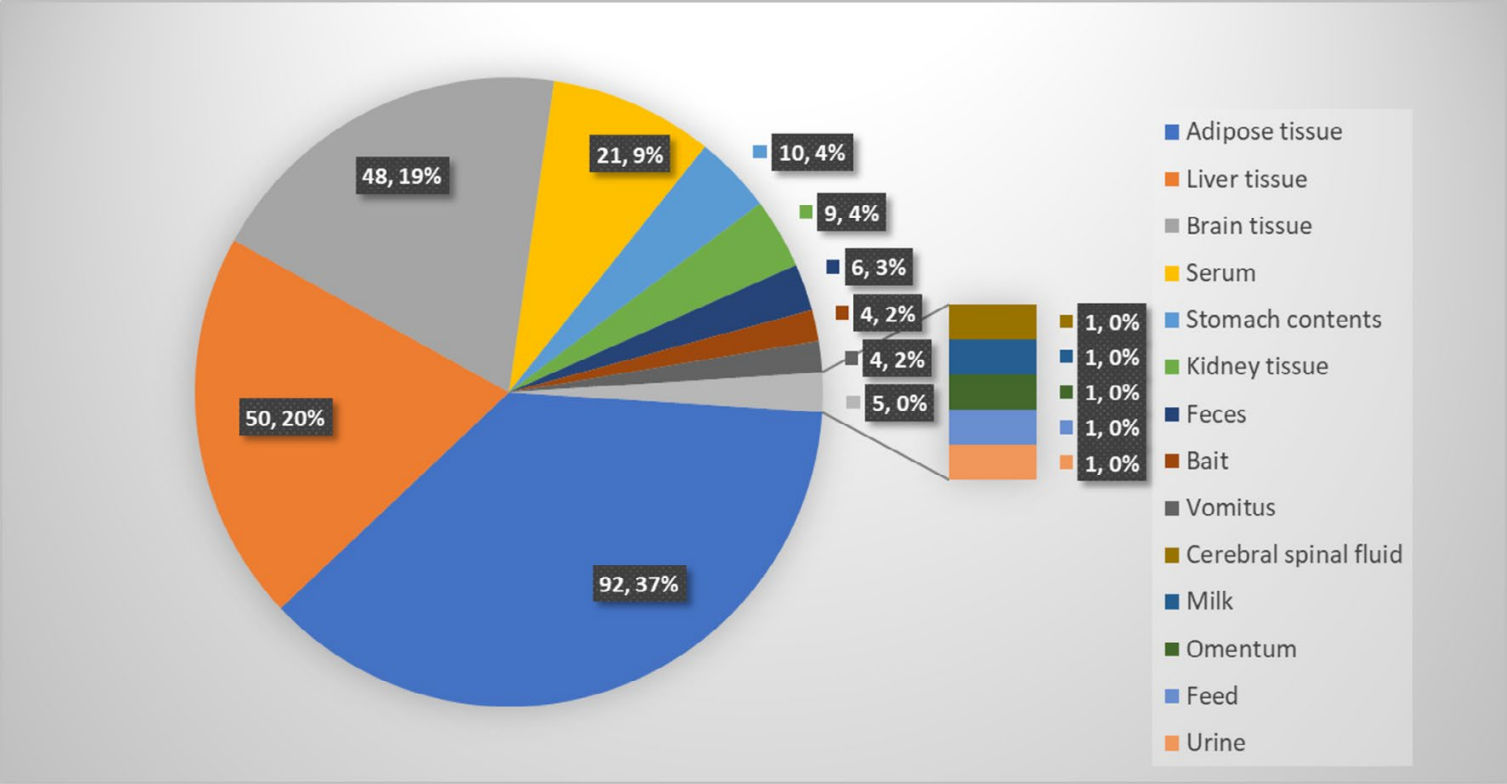
Distribution of the specimens that were evaluated for bromethalin at the Toxicology Section of the California Animal Health and Food Safety Laboratory between 2017 and 2023.

The most common samples submitted for analysis included adipose tissue (92 samples [37%]; 36 from dogs [27%] and 56 from cats [49%]), liver tissue (50 samples [20%]; 20 from dogs [15%] and 30 from cats [26%]), brain tissue (48 samples [19%]; 31 from dogs [24%] and 17 from cats [15%]), serum (21 samples [9%]; 14 from dogs [11%] and 7 from cats [6%]), and stomach contents (10 samples [4%]; 9 from dogs [7%] and 1 from a cat [1%]). Additional miscellaneous samples included feces, bait, vomitus, cerebrospinal fluid, milk, omentum, and urine (Figure 5).

**FIGURE 6:**
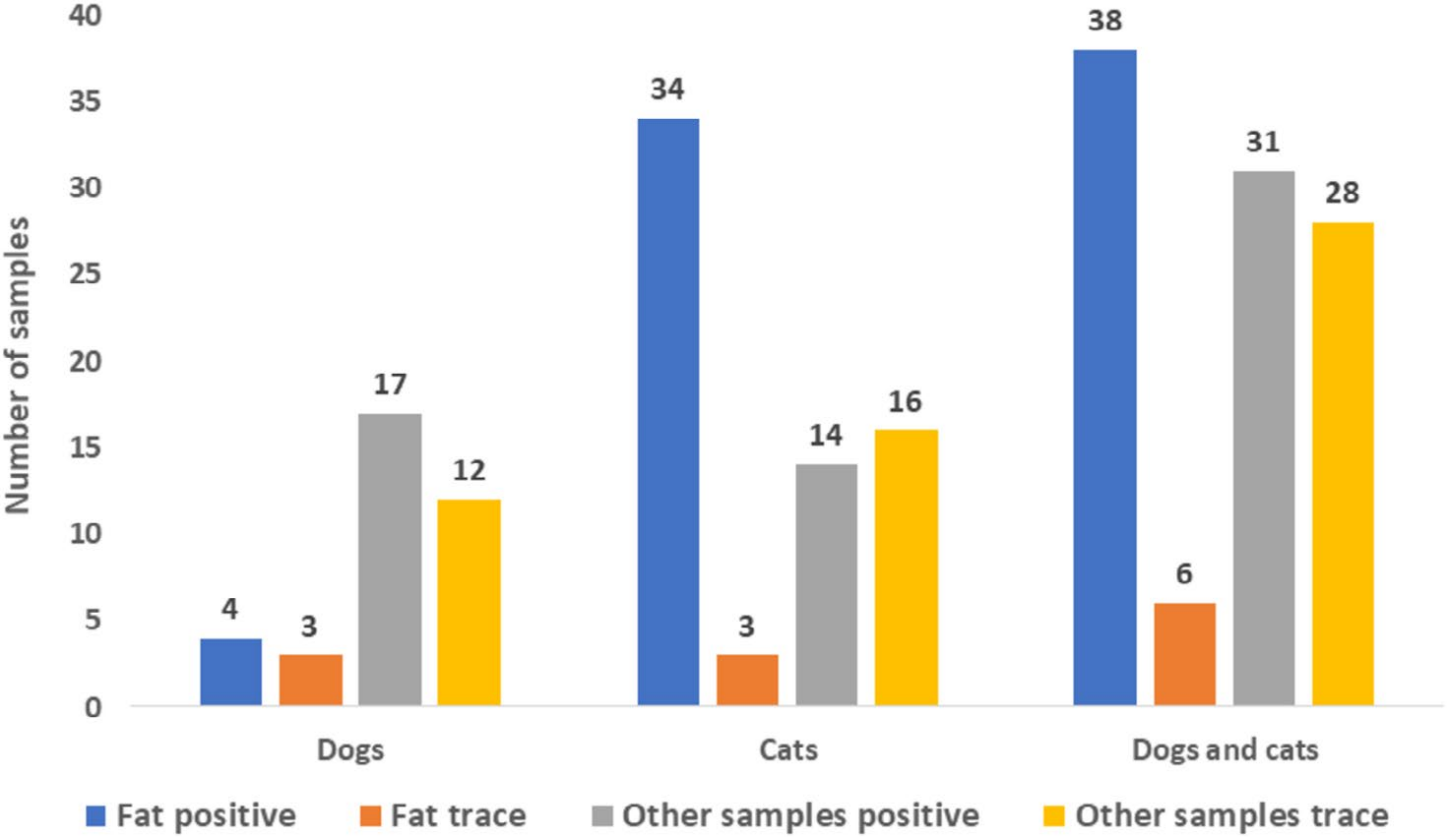
Comparison of positive and trace bromethalin analysis results between adipose tissue and other tissues combined, in dogs, cats, and all samples.

Table 1 summarizes the samples submitted for analysis across the 60 positive cases (43 cats and 17 dogs) and 31 trace cases (17 cats and 14 dogs). Among all cases and among cats, fat tissue had significantly more positive results compared to other tissues (*p* = 0.0002), while other tissues had more trace results. (Figure 6).

**TABLE 1:** Samples submitted for analysis across the 60 positive cases (43 cats and 17 dogs) and 31 trace cases (17 cats and 14 dogs).

Category	Adipose tissue	Serum	Liver	Brain	Omentum	Kidney	Stomach content
Overall positive cases (*n* = 60)	40 (67%)	12 (20%)	3 (5%)	2 (3%)	1 (2%)	1 (2%)	1 (2%)
Positive cases—cats (*n* = 43)	32 (74%)	5 (12%)	3 (7%)	2 (5%)	1 (2%)	—	—
Positive cases—dogs (*n* = 17)	8 (47%)	7 (41%)	—	—	—	1 (6%)	1 (6%)
Overall trace cases (*n* = 31)	8 (26%)	1 (6%)	9 (29%)	7 (23%)	—	1 (6%)	5 (16%)
Trace cases—cats (*n* = 17)	3 (18%)	—	8 (47%)	5 (29%)	—	—	1 (6%)
Trace cases—dogs (*n* = 14)	5 (36%)	1 (7%)	1 (7%)	2 (14%)	—	1 (7%)	4 (29%)

Eighteen cases involved the submission of more than one sample for analysis. In 8 cases, all results were negative. In 10 cases, at least a trace amount of DMB was detected in at least one sample. Of these, adipose tissue was submitted in 8 cases, and DMB was detected in all (with or without detectable levels in other samples, including brain, liver, urine, and stomach content). In one case, both serum and milk contained trace amounts of DMB; in one case, both bait and stomach contents had detectable and trace amounts of DMB, respectively.

In none of the negative cases was there a known exposure to the toxin. However, in 13 of the confirmed DMB exposure cases, there was a known exposure (12 out of 31 dogs and 1/60 cats).

The duration of illness at presentation was reported for only 56 cases of all submitted cases: 31 dogs and 25 cats (overall median 2 days, range 2.5 h to 17.5 days; Dogs: median 1.5 days, range 2.5 h to 14 days; Cats: median 4 days, range 2.5 h to 17.5 days) and was defined as “acute” in 16 cases (13 dogs and 3 cats). The duration of illness at presentation was reported for only 30 cases with confirmed exposure to DMB (positive or trace): 13 dogs and 17 cats (overall median 3 days, range 18 h to 17.5 days; Dogs: median 2 days, range 18 h to 4 days; Cats: median 5 days, range 1 day to 17.5 days) and was defined as “acute” in 6 cases (4 dogs and 2 cats). The duration of illness at presentation was significantly longer in cats compared to dogs in cases with confirmed exposure to bromethalin (*p* = 0.028).

Overall, only 70 cases (21 dogs and 49 cats) included medical history information and/or clinical signs on the case submission form. The terminology used by the referring doctors was inconsistent, likely due to the involvement of multiple referral clinics and labs from 35 different states. Therefore, the history and clinical signs were categorized into general groups. In some cases, more than one clinical sign was reported, and in some cases, there was a progression from one condition to another. All clinical signs were summarized accordingly (Table 2).

**TABLE 2:** Clinical signs reported on the case submission form for 49/60 trace/positive cats and 21/32 trace/positive dogs.

Clinical signs	Dogs (*n* = 21)	Dogs (%)	Cats (*n* = 49)	Cats (%)
Neurological signs	3	14%	14	29%
CNS signs				
Seizures	6	29%	2	4%
Altered mentation	6	29%	15	31%
Tremors	1	5%	6	12%
Hyperesthesia	1	5%	—	—
Myelopathic nervous system signs				
Recumbency	4	19%	11	23%
Weakness	3	14%	6	12%
Ataxia	3	14%	12	25%
Paresis/paralysis	3	14%	9	18%
Rigidity	1	5%	6	12%
CP deficits	—	—	6	12%
Other signs				
Green feces	4	19%	1	2%
Found dead/dead on arrival	2	10%	5	10%

Abbreviations: CNS, central nervous system; CP, conscious proprioception.

Out of 21/32 trace and positive dogs with relevant reports, 3 (14%) were reported to suffer from “neurological” signs. Regarding CNS signs, 6 (29%) had seizures, 6 (29%) experienced altered mentation, 1 (5%) had tremors, and 1 (5%) exhibited hyperesthesia. Myelopathic nervous system signs included recumbency in 4 (19%) cases, weakness in 3 (14%) cases, ataxia in 3 (14%) cases, paresis/paralysis in 3 (14%) cases, and rigidity in 1 (5%) case. In 4 (19%) cases, green feces were noted, and 2 (10%) dogs were either found dead or were dead on arrival.

Out of 49/60 trace and positive cats with relevant reports, 14 (29%) were reported to suffer from “neurological” signs. Regarding CNS signs, 15 (31%) experienced altered mentation, 6 (12%) had tremors, 5 (10%) were blind, and 2 (4%) experienced seizures. Myelopathic nervous system signs included ataxia in 12 (25%) cases, recumbency in 11 (23%) cases, paresis/paralysis in 9 (18%) cases, weakness in 6 (12%) cases, decerebrate posture or rigidity in 6 (12%) cases, and conscious proprioception deficits in 6 (12%) cases. In one case (2%), green feces were noted, and 5 (10%) cats were either found dead or were dead on arrival.

Seventeen cases (11 cats and 6 dogs) reported MRI results, of which 3 cases (2 cats and 1 dog) were negative for DMB. The MRI findings in the negative cases were described as follows: for the cats, “hyperintensity” and “post‐mortem MRI—cytotoxic edema/intramyelinic edema suggestive of bromethalin intoxication,” and for the dog, “diffuse white matter lesions.”

In the trace/positive feline cases, one MRI was considered unremarkable, while four scans indicated results “consistent with bromethalin toxicosis” (with one being a post‐mortem MRI). Additionally, four cases reported findings such as white matter edema (brain and spinal cord); T2 + flair symmetrically bilateral white matter hyperintensity with changes on DWI/ADC; diffuse white matter tract hyperintensity with restricted diffusion on DWI; and diffuse white matter edema in the brain. All these cases included remarks indicating “consistent with cytotoxic edema” or “consistent with bromethalin toxicosis.” In the trace/positive canine cases, one dog was reported as “abnormal on MRI,” and four cases were described as having: diffuse white matter lesions; diffuse and symmetric T2 hyperintensity throughout the cerebral white matter and ventral pons; diffuse bilaterally symmetric white matter changes; abnormal increase in signal intensity in cortical white matter, bilateral in distribution, hyperintensity on T2‐weighted images, hyperintensity on DWI, and hypointensity on ADC images; diffuse uniform white matter cytotoxic/intramyelinic edema; and white matter cytotoxic edema with bilaterally symmetric changes consistent with toxic or metabolic causes.

Post‐mortem results were available for 33 trace/positive cases (8 dogs and 25 cats). Among the 4 dogs with a trace amount of DMB, 3 did not exhibit any relevant findings on autopsy, while 1 dog had green bait present throughout the gastrointestinal tract and in the feces. Among the 4 positive dogs, findings included no lesions in one dog, while another exhibited green‐blue bait in the distal colon/rectum and possible unspecified brain lesions. Diffuse deep laminar cortical edema and cerebral edema were observed in 2 dogs. Of the 8 cats with a trace amount of DMB, one exhibited brain vacuolization, which the pathologist questioned as potentially resulting from autolytic changes. The remaining 7 cats showed varying degrees of diffuse, mild to severe white matter spongy degeneration and edema in the brain and spinal cord, as well as vacuolar myelopathy. Among the 17 positive cats, findings included varying degrees of diffuse, mild to severe white matter spongy degeneration, edema, vacuolar myelopathy, and demyelination in the brain and spinal cord. Additionally, flattened gyri with cerebral edema were observed in 3 cases, pulmonary congestion and edema in 6 cases, and blue‐green material in the stomach and descending colon in 1 case.

One hundred ninety/223 animals were dead at the time the samples were submitted. Thirteen were alive at the time of submission, and in 21 cases, the status was unknown.

## 4 Discussion

In 1988, the EPA began reviewing the safety of rodenticides introduced before the agency's formation. A decade later, the EPA proposed limiting second‐generation ARs to professional and some agricultural use only, banning them from household consumer use. This restriction was finalized in 2008. Since then, there has been a rise in human and animal exposure to bromethalin [18], as evidenced by the toxicology laboratory at the California Animal Health and Food Safety Laboratory data and the 2.8‐fold increase in submitted cases from 2010–2016 to 2017–2023 (from 59 to 164 cases, respectively).

In assessing demographic features, 25% more dog cases than cat cases were submitted for analysis. There was no significant difference between males and females in either species. The median age of dogs was 3 years, consistent with previous publications [12]. Cats were significantly younger than dogs, with a median age of 2 years. Cats are traditionally considered less prone to poisoning than dogs [19], which could explain the difference in submissions and the age gap. Younger cats might be more curious, less experienced, and less cautious, leading them to consume the bait, while dogs tend to be less cautious than cats at any age. Another finding was that cats were significantly more likely to have evidence (trace or positive) of DMB exposure than dogs. This might be related to the fact that dogs are more often observed consuming toxicants (e.g., bromethalin baits), reducing the need for laboratory confirmation of bromethalin ingestion [12]. In contrast, cats that are indoors/outdoors or solely outdoors are usually not closely monitored by their owners; thus, ingestion might occur unnoticed. It is also possible that cats are simply more exposed to bromethalin because it is found more often in barns and other habitats where cats reside.

Adipose tissue is considered the preferred sample for confirming or ruling out bromethalin exposure or intoxication, as it can be easily collected from both live and deceased animals [20]. In the present study, adipose tissue was the sample of choice, followed by liver and brain tissues. Serum was tested in 9% of the cases and was found to be either positive or trace in 13 out of 21 samples. In one case, milk from a dog that ingested a 1‐oz bar of 0.01% bromethalin while nursing 12 puppies, 4 days old, was submitted and found to contain trace amounts of bromethalin. In this instance, emesis was induced within 1 h of ingestion, activated charcoal was administered, and the dog did not exhibit clinical signs. Serum from the dog was also tested and trace amounts of bromethalin were detected. Unfortunately, the case was lost to follow‐up, so no information is available regarding the puppies. To the best of our knowledge, there are no published data on the excretion of bromethalin in milk, making this the first evidence that it can be present in milk. Additionally, in the present study, samples from 14 kittens and 3 puppies, assessed to be 2 months old or younger, were submitted. Of these, 14 tested positive for DMB, and 3 had trace amounts of DMB. Five of the kittens were estimated to be 3 weeks old (and therefore expected to be exclusively suckling), with one found nursing from a deceased queen. This raises a concern that bromethalin can pass into milk and pose a hazard to suckling litters.

In most of the positive cases, the test was conducted on adipose tissue, whereas in the majority of trace result cases, other tissues were used. This might suggest that if adipose tissue had been tested in some of the trace cases, the results might have been positive. Given that bromethalin is lipophilic and accumulates in high concentrations in fat tissue, using adipose tissue for testing could potentially increase the likelihood of detecting the toxicant. Moreover, in 18 cases, more than one sample per case was submitted. In 8 cases, all results were negative, while in 10 cases, at least a trace amount of DMB was detected in at least one sample. When adipose tissue was submitted along with other samples, DMB was always present in the fat whenever there was evidence of it in any sample, even if other samples were negative (Table 1). This finding supports the conclusions of Bautista et al. [20], Filigenzi et al. [17], and Romano et al. [21], suggesting that adipose tissue is the tissue of choice for confirming or ruling out bromethalin exposure or intoxication. Serum might also serve as a good sample material, but more studies are needed.

Based on medical history, in all of the witnessed bromethalin exposure cases, there was a confirmed toxicological test of exposure (trace or positive). In contrast, none of the negative cases had a known exposure. This “field‐based” data supports the accuracy of the LC–MS/MS method for analyzing DMB in tissue samples [17].

The duration of illness before the submission of samples for testing was significantly longer for cats compared to dogs. This finding could be biased by the fact that dog owners often witness their pets consuming toxicants [12], so toxicology tests are initiated more quickly. Additionally, clinical signs in dogs are often more aggressive and acute, with the “convulsant syndrome,” prompting faster action. In contrast, cats are usually not seen consuming the poison and tend to develop the “paralytic syndrome” regardless of dosage, with a slower onset and more subtle clinical signs, leading to a longer period before intoxication is suspected.

The clinical signs reported in the present study are consistent with those in the literature [3, 5, 7, 8, 11, 12]. Cats tended to suffer more from myelopathic signs, consistent with the “paralytic syndrome.” However, 31% of cats for whom there was a report, also demonstrated altered mentation. Dogs, on the other hand, exhibited both syndromes: “paralytic and convulsant syndromes”, 29% for whom there was a report experiencing seizures and 29% showing altered mentation.

Seventeen cases submitted for bromethalin testing included MRI results. To the best of our knowledge, MRI findings in bromethalin‐poisoned animals have been described in only two case reports (involving two cats and three dogs) [15, 16], and in one study on MRI pattern recognition of metabolic encephalopathies, which included one cat with bromethalin toxicosis [22]. Suggesting that MRI has the potential to be a valuable diagnostic tool for the antemortem diagnosis of bromethalin intoxication in dogs and cats.

Characteristic MRI findings typically show marked hyperintensity of the brain and spinal cord white matter on T2‐weighted sequences, indicative of edema. DWI might reveal restricted diffusion and hyperintensity within white matter structures, while ADC maps might demonstrate corresponding hypointensity, consistent with intramyelinic edema [15, 16, 22]. The areas most often affected include the corpus callosum, corona radiata, internal capsule, and corticospinal motor tracts [22]. These MRI findings are consistent with diffuse leukoencephalopathy and have been observed in both dogs and cats. In one reported case, the resolution of MRI abnormalities in a surviving dog correlated with the resolution of clinical signs [16]. In some cases, severe cerebral edema might result in the effacement of normal cerebral and cerebellar sulci [22], with lesions extending from the forebrain to the cerebellum and along the pyramidal tracts of the medulla oblongata [22].

Among the 17 cases, three were negative for DMB but had MRI lesions that the referring veterinarian deemed consistent with bromethalin exposure. In one of these cases, the MRI was conducted postmortem. Of the remaining cases that suggested bromethalin exposure (either trace or positive), one had an unremarkable MRI, while others had MRI reports describing findings “consistent with cytotoxic edema or bromethalin toxicosis” or offering specific descriptions supporting the diagnosis. The MRI lesions in the three DMB‐negative cases could potentially be explained by global cerebral ischemia leading to cytotoxic edema, unrelated to bromethalin toxicosis in one case in which the MRI was done post‐mortem [23]. In the 2 other cases, differential diagnoses for the MRI changes could include other toxic and metabolic causes, such as carbon monoxide, organotin compounds, hexachlorophene, hypoglycemia, nutritional deficiencies, age‐associated periventricular lesions, and leukocentric presentations of infectious or immune‐mediated diseases [15, 16, 22]. Notably, one cat with “unremarkable” MRI results had a trace amount of DMB detected, and the autopsy confirmed white matter edema in the brain and spinal cord on histology. The fact that 13 out of 17 cases (77%) that underwent MRI showed findings consistent with confirmed bromethalin exposure suggests that MRI is a promising supporting antemortem diagnostic tool and warrants further investigation.

The majority of information regarding bromethalin intoxication is derived from administration studies that describe postmortem lesions [7, 24]. In these experimental models, dogs were administered 6.25 mg/kg and cats 1.5 mg/kg of bromethalin—dosages that significantly exceed the minimum lethal doses (2.5 mg/kg in dogs [7], and 0.45 mg/kg in cats [8]). The characteristic histopathological lesion observed in bromethalin‐exposed animals was a diffuse, vacuolar spongiosis of the white matter within the cerebellum, cerebrum, brainstem, spinal cord, and optic nerve. There are only scarce clinical case reports and case series describing CNS lesions associated with bromethalin toxicosis in mammals [15, 16, 20, 21, 25, 26, 27], with most highlighting histopathological findings consistent with those observed in experimental models. One case series reports fatal bromethalin intoxication in three cats and two dogs with minimal or no histologic CNS changes [21]. In the present study, postmortem results were available for 33 cases with trace or positive DMB levels. None of the four dogs diagnosed with trace amounts of DMB had histologic lesions in the CNS (though one had green bait present in the GI tract). Of the four dogs with positive DMB levels, only two showed CNS lesions that could be compatible with bromethalin toxicosis. In contrast, in the cat group, all but one cat demonstrated histopathological CNS lesions that might be compatible with bromethalin toxicosis. The lack of histopathological findings consistent with bromethalin toxicosis in some cases might be explained by the lower doses of bromethalin consumed in clinical cases compared to those used in experimental studies. This aligns with observations that white matter spongiform degeneration was not present in rats administered lower doses of bromethalin [6]. The absence of histological findings in dogs with only trace amounts of DMB in our study could support this explanation. Another factor to consider is the timing from ingestion to death, which could influence the histological appearance of white matter. All cats that underwent postmortem examination, except one where autolysis was too severe, showed evidence of spongy degeneration. In contrast, most dogs did not exhibit classic CNS white matter spongiform changes. Notably, the duration of illness at presentation was significantly longer in cats than in dogs, which might also support this hypothesis. More studies are needed to validate these findings, ideally using quantitative bromethalin measurement methods.

This study has a few inherent limitations. First, the retrospective nature of the study and the fact that it was based on information from referral forms rather than comprehensive medical records led to missing data. Moreover, cause‐and‐effect relationships often cannot be determined retrospectively. Second, the cohort size, although the largest yet, is nonetheless limited, which weakens the descriptive information. Third, the results of the bromethalin test at the toxicology laboratory of the California Animal Health and Food Safety Laboratory are qualitative rather than quantitative. For diagnostic purposes, this does not change the clinical course of the case since proof of exposure to bromethalin is unequivocal. However, exposure does not necessarily confirm intoxication. It would have been interesting to compare DMB concentrations to morbidity and mortality. Fourth, this study comprised clinical data from a single referral veterinary toxicological laboratory. Therefore, our results should be applied cautiously to other clinical settings.

In conclusion, the restriction on using ARs has led to increased human and animal exposure to bromethalin, resulting in a rise in toxicology samples submitted for analysis. Twenty‐five percent more dog cases were analyzed compared to cats, yet cats were significantly younger and more likely to test positive or show trace amounts of DMB. Adipose tissue remains the preferred sample for confirming or ruling out bromethalin exposure, though serum might also be a useful option. There is some evidence suggesting that bromethalin might pass into milk, which requires further investigation. Cats experienced a significantly longer duration of illness before testing than dogs. Seventy‐seven percent of the cases that underwent MRI showed findings consistent with bromethalin exposure, suggesting that MRI might be a promising supporting antemortem diagnostic tool and warranting further investigation. In postmortem examinations, all but one cat exhibited spongy degeneration, whereas most dogs did not show the classic CNS white matter spongiform changes.

## Disclosure

Authors declare no off‐label use of antimicrobials.

## Ethics Statement

Authors declare no Institutional Animal Care and Use Committee or other approval was needed. Authors declare human ethics approval was not needed.

## Conflicts of Interest

The authors declare no conflicts of interest.

## Supporting information


**Data S1.** Supporting Information.
