# Supplementary material for: Bromethalin Exposure in Dogs and Cats: A 14‐Year Retrospective Study (2010–2023) From the California Animal Health and Food Safety Laboratory System
Source: J Vet Intern Med. 2025 Mar 26;39(3):e70057. doi: 10.1111/jvim.70057 (PMC11938294; doi:10.1111/jvim.70057)
Supplement: Supplementary file 1 — Data S1. Supporting Information. [file JVIM-39-e70057-s001.docx]

Materials and methods

*Analytical method for detection of DMB*

The analysis to detect bromethalin exposure utilized at “BLINDED FOR REVIEW” is based on a published and validated method and relies on the detection of the metabolite, DMB, in tissues or serum by LC-MS-MS.16 Briefly, tissue samples are extracted by homogenizing 0.5g of tissue sample (adipose, brain, liver) with 10 mL of ethyl acetate with a Genogrinder (model 2010, SPEX SamplePrep, Metuchen, NJ, USA). The sample is then centrifuged and evaporated under nitrogen at ~40°C (N-EVAP model 112, Organomation Associates Inc, Berlin, MA, USA). The dry extract is reconstituted with 5mL of acetonitrile, vortexed for ~30 seconds, and immediately transferred through two rounds of lipid cleanup using EMR-Lipid dispersive solid phase extraction (Agilent Technologies, Santa Clara, CA, USA). The acetonitrile is then evaporated under nitrogen at ~40°C, reconstituted in 250µL of methanol, and filtered through a 0.22µm PES syringe filter (Sigma Aldrich, St. Louis, MO, USA) into an autosampler vial with 300µL insert and submitted for analysis. Approximate extraction time including evaporating steps is 1.5-2 hours. Samples are analyzed on an Infinity 1290 HPLC (Agilent Technologies, Santa Clara, CA, USA) coupled to a Sciex 6500 or 7500 triple quadrupole Q trap mass spectrometer (Sciex, Carlsbad, CA, USA). The acquisition time is 19 minutes. The HPLC column is a 100 mm × 2.1 mm i.d., 1.7 μm, Zorbax Eclipse Plus C 18 column fitted with an Eclipse Plus guard column (Agilent). Mobile phases consist of an aqueous phase (0.1% formic acid in water) and an organic phase (0.1% formic acid in acetonitrile). The flow rate is 0.35mL/min. An LC gradient starting at 20% organic is increased to 95% organic at 6 min and held for 7 min then switched back to 20% organic and held for 4 min. The mass spectrometer is set to MRM scan mode and negative polarity at a spray voltage of 4500V, source temperature at 400°C, curtain gas at 40psi, ion source gas 1 at 70psi, ion source gas 2 at 75psi. The DMB precursor mass is m/z 562 and the fragment ions are m/z 254 (EP -10, CE -41, CXP -5) and m/z 278 (EP -10, CE -35, CXP -5). The analytical sequence consists of a DMB standard at the beginning and end of the sequence, and a matrix spike and matrix blank before the sample. The matrix spike is fortified at the reporting limit for the analysis. If a signal for DMB is detected at a concentration greater than that of the reporting limit spike, the sample is declared positive for DMB. If a signal is detected but is below the reporting limit spike and at least 3x the amount of any background noise, the sample is declared “trace” for DMB. If no signal is detected, the sample is declared non-detect for DMB.
